# From hormones to neurodegeneration: how FSH drives Alzheimer’s disease

**DOI:** 10.3389/fnagi.2025.1578439

**Published:** 2025-06-16

**Authors:** Yafei Xue, Shuqi Zuo, Fei Wang, Xiaoyi Qi

**Affiliations:** Department of Obstetrics and Gynaecology, Shandong Provincial Hospital Affiliated to Shandong First Medical University, Jinan, China

**Keywords:** follicle-stimulating hormone, Alzheimer’s disease, aging, FSH-blocking antibodies, neuroinflammation, lipid accumulation

## Abstract

The role and function of follicle-stimulating hormone in the gonads have been extremely studied. However, recent research has begun to explore the relationship between elevated follicle-stimulating hormone levels and the prevalence of extragonadal disorders, particularly in perimenopausal and postmenopausal women. These disorders include endometrial cancer, osteoporosis, obesity, and atherosclerosis. This review provides new insights into the relationship between follicle-stimulating hormone and the development of age-related diseases, with a focus on Alzheimer’s disease. Follicle-stimulating hormone does not act alone in promoting Alzheimer’s disease but often works in conjunction with inflammation, lipid accumulation, and vascular alterations. Furthermore, follicle-stimulating hormone synergizes with obesity, gut microbiota, autophagy, and aging, creating conditions that facilitate the onset and progression of Alzheimer’s disease. This review also summarizes the therapeutic potential of FSH-blocking antibodies in treating these diseases.

## 1 Introduction

Perimenopausal and postmenopausal women are at high risk for various age-related diseases, including cardiovascular diseases (Hodis and Mack, 2022), musculoskeletal symptoms (Wright et al., 2024) and cognitive dysfunction (Mosconi et al., 2021). Hormonal changes, particularly the decline in estrogen levels, are closely associated with these risks. As women transition from perimenopause to postmenopause, estrogen levels drop significantly and remain low. However, estrogen replacement therapy remains controversial, with some studies reporting no improvement or even worsening of cognitive function (Cheng et al., 2021). In contrast, follicle-stimulating hormone (FSH) levels begin to rise approximately 2 years before the final menstrual period (FMP) and stabilize 2 years after the FMP, often remaining elevated for decades (Wang et al., 2021). This trend parallels the onset of age-related diseases.

FSH is a gonadotropin secreted by the anterior pituitary gland that binds to FSH receptors (FSHRs), which belong to the class A/rhodopsin subfamily of G protein-coupled receptors. Traditionally, FSH was thought to act primarily on gonadal tissues, specifically sertoli cells in the testes and granulosa cells in the ovaries. However, recent evidence suggests that FSH also plays a role in extragonadal diseases (Table 1).

**Table 1:** Follicle-stimulating hormone receptor (FSHR) expression in extragonadal tissues.

Type of tissue	Sample size	Main methodology	Observation	Reference
Osteoclasts	Mice (*n* = 4-14/group)	RT-PCR, WB, FACS (Anti-FSHR Ab: Thermo), IF	FSHR mRNA (*p* < 0.05) and protein expressed in osteoclasts and precursors	Sun et al. (2006)
Osteoclasts	Human (*n* = NA)	RT-PCR, WB	FSHR mRNA and protein expressed in osteoclasts	Sun et al. (2006)
Vein endothelial cells	Human cell line	WB (Anti-FSHR Ab: Proteintech 22,665-1-AP), IF	FSHR protein expression in Human umbilical vein endothelial cells	Tan et al. (2021)
Prostate tumor vascular endothelial cells	Human (*n* = 773)	IHC (Anti-FSHR Ab: FSHR 323, FSHR190, FSHR225)	Prostate tumor vascular endothelial cells are positive for FSHR relative to normal tissue	Radu et al. (2010)
Liver	Human (*n* = NA)	RT-PCR, WB (Anti-FSHR Ab: Proteintech, Abcam), IF	Expression and localization of FSHR in human liver	Guo et al. (2019)
Liver	Mice (*n* = NA)	RT-PCR, WB (Anti-FSHR Ab: Proteintech, Abcam), IF (Anti-FSHR Ab: Proteintech)	Expression and localization of FSHR in mouse liver	Guo et al. (2019)
Adipocytes	Human (*n* = NA)	RT-PCR, WB (Anti-FSHR Ab: Abcam), IF, IHC	Expression of FSHR in human adipocytes	Liu et al. (2015)
Adipocytes	Mice (*n* = NA)	RT-PCR, WB (Anti-FSHR Ab: Abcam), IF	Expression of FSHR in mouse adipose tissue	Liu et al. (2015)
Endometrial cancer	Human (*n* = 34), Human cell lines	WB (Anti-FSHR Ab: Abcam ab150557), IHC (Anti-FSHR Ab: Abcam ab150557), IF (Anti-FSHR Ab: Abcam ab113421)	FSHR protein expression in endometrial cancer (p < 0.05)	Sheng et al. (2022)
Pancreas	Rat (*n* = 5)	IF (Anti-FSHR Ab: Santa Cruz), IHC (Anti-FSHR Ab: Santa Cruz)	FSH receptor mainly located in some islet cells	Chu et al. (2010)
Brain	Human (*n* = NA)	RT-PCR	FSHR expression in cortex and neuroblastoma cells	Xiong et al. (2022)
Brain	Mice (*n* = NA)	RT-PCR, WB (Anti-FSHR Ab: Thermo PA5–50963)	FSHR expression in cortex and hippocampus	Xiong et al. (2022)
Brain	Rat (*n* = NA)	RT-PCR	FSHR expression in cortical neurons	Xiong et al. (2022)

RT-PCR, reverse transcription PCR; WB, Western blotting; FACS, Fluorescence-Activated cell sorting; NA, not applicable; IHC, immunohistochemistry.

Alzheimer’s disease (AD) is a degenerative brain disorder and the leading cause of dementia worldwide. Perimenopausal and postmenopausal women are particularly susceptible to AD. According to the American Alzheimer’s Association in 2020, the prevalence of AD increases dramatically with age (Alzheimer's disease facts and figures, 2022), and women are disproportionately affected compared to men. While age is a significant factor, women live longer than men, they are more prone to developing brain lesions, thus increasing the prevalence of AD. The onset of AD is also strongly associated with elevated serum FSH levels. Studies have shown that postmenopausal women with AD have higher serum FSH concentrations than their healthy counterparts, independent of estrogen levels. Recent research has demonstrated that FSH affects neurons, establishing it as an AD-promoting hormone (Xiong et al., 2022). FSHR expression has been detected in the human cortex, neuroblastoma cells (SH-SY5Y), mouse cortex, hippocampus, and rat neurons (Table 2). Knockdown of hippocampal FSHR has been shown to improve AD neuropathology and spatial memory impairment (Xiong et al., 2022).

**Table 2:** Follicle-stimulating hormone receptor (FSHR) expression in nerve cells.

Sample size	Methodology	FSHR expression	Reference
Rat (*n* = 6)	IF (Anti-FSHR Ab: Santa Cruz), IHC (Anti-FSHR Ab: Santa Cruz)	FSHR positive signals were located in hippocampus	Chu et al. (2008)
Human (*n* = NA)	RT-PCR	FSHR expression in cortex and neuroblastoma cells	Xiong et al. (2022)
Mice (*n* = NA)	RT-PCR, WB (Anti-FSHR Ab: Thermo PA5–50963)	FSHR expression in cortex and hippocampus	Xiong et al. (2022)
Rat (*n* = NA)	RT-PCR	FSHR expression in cortical neurons	Xiong et al. (2022)
Rat (*n* = 6)	IF (Anti-FSHR Ab: Santa Cruz)	FSHR positive signals were located in cerebellar cortex	Chu et al. (2013)

IHC, immunohistochemistry; NA, not applicable; RT-PCR, reverse transcription PCR; WB, Western blotting.

## 2 Role of FSH in Alzheimer’s disease

AD is pathologically characterized by amyloid plaques formed by amyloid-*β* (Aβ) peptides and neurofibrillary tangles (NFTs) composed of hyperphosphorylated Tau proteins. These pathological features lead to synaptic loss, neuronal degeneration, and the hallmark symptoms of AD: memory impairment, cognitive decline, and behavioral dysfunction (Fedele, 2023). The amyloid cascade hypothesis remains the dominant theory explaining AD pathogenesis. According to this hypothesis, A*β* peptides are produced through the cleavage of amyloid precursor protein (APP) by β-secretase and *γ*-secretase, resulting in various Aβ isoforms, including Aβ42, which is considered the primary driver of AD (Zhang Y. et al., 2023). Dysregulation of Aβ production or clearance leads to its accumulation and aggregation into soluble oligomers and insoluble fibrils. Tau, a protein predominantly found in neuronal axons, plays a critical role in neurite outgrowth, cell shape, and intracellular transport. Hyperphosphorylated Tau disrupts protein degradation systems, such as the ubiquitin-proteasome and phagosome-lysosome pathways, leading to the accumulation of waste proteins in neurons (Drummond et al., 2020).

**Figure 1:**
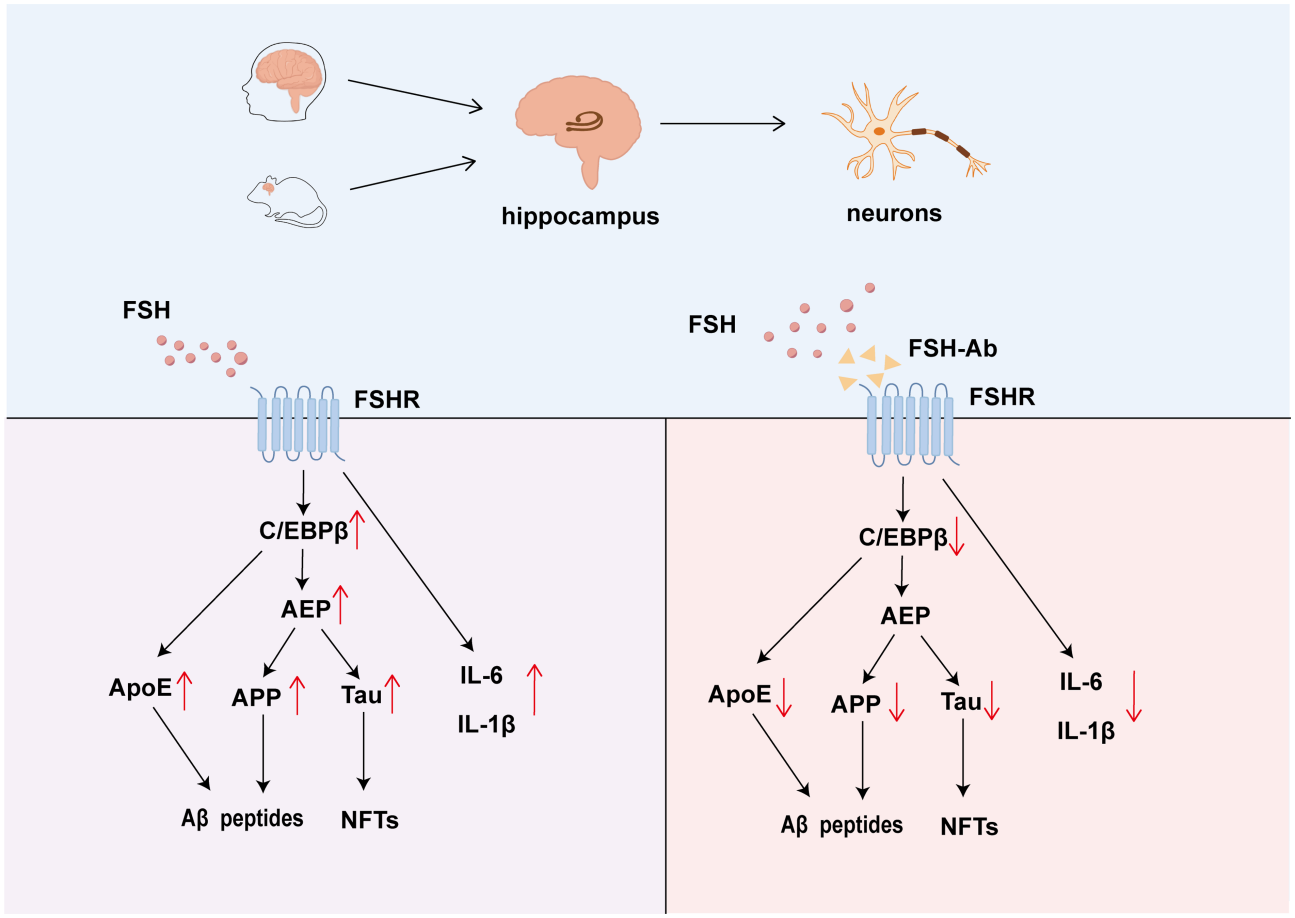
The role of FSH-FSHR signaling in neurons and hippocampus. FSH promotes the formation of amyloid plaques through Aβ peptide aggregation and the development of NFTs. Additionally, FSH triggers the release of ApoE via the C/EBPβ–*δ*-secretase pathway, which further elevates levels of IL-6 and IL-1β. The action of FSH is antagonized by FSH-Ab, which binds to FSHR and inhibits it’s signaling.

Xiong et al. (2022) confirmed that FSHR is expressed in the cortex, hippocampus, and neuronal cells by end-point polymerase chain reaction (PCR) or immunofluorescence staining (IF). IF revealed that FSH triggers the expression of C/EBPβ, arginine endopeptidase (AEP), cleaved APP, Tau proteins, Aβ40, and Aβ42 in mice. Consistent with these molecular changes, Morris water maze testing demonstrated that FSH-injected mice exhibited spatial memory impairment, indicating FSH-induced cognitive decline. In ovariectomized mice, hippocampal FSHR knockdown reduced the expression of C/EBPβ, AEP, cleaved APP, and Tau, ameliorating AD neuropathology and spatial memory impairment, independent of estrogen. This was further supported by Golgi staining, transmission electron microscopy and Morris water maze testing. Additionally, FSHR activation in human SH-SY5Y cells and primary rat neuronal cells induced amyloidogenic protein accumulation and the release of inflammatory cytokines IL-6 and IL-1β. These findings confirm the direct role of FSH in AD through the C/EBPβ–*δ*-secretase pathway and provide a basis for targeting FSH in AD treatment (Figure 1).

## 3 Potential mechanisms by which FSH increases risk of AD

FSH contributes to AD pathogenesis both directly and in tandem with other mechanisms, including inflammation, lipid accumulation, and vascular alterations.

### 3.1 Inflammation

Neuroinflammation is a key factor in AD pathogenesis. Chronic neuroinflammation, driven by glial overactivation, is considered the third core pathological feature of AD, alongside Aβ plaques and NFT (Leng and Edison, 2021). When the balance between pro-inflammatory and anti-inflammatory signals is disrupted, glial cells release interleukin (IL)-1β and tumor necrosis factor (TNF)-*α*, leading to neuronal damage through excessive phagocytosis (Twarowski and Herbet, 2023). Inflammatory cytokines also exacerbate Aβ accumulation and Tau propagation (Chen and Yu, 2023). Xiong et al. (2022) demonstrated that FSH increased the expression of IL-1β (1.4-fold, *p* < 0.001) and IL-6 (1.75-fold, *p* < 0.001) in human primary neuronal cells, along with elevated levels of APP and Tau. FSHR knockdown reduced the expression of these inflammatory markers, suggesting that FSH promotes neuroinflammation and exacerbates AD pathology (Xiong et al., 2022).

Systemic inflammation, triggered by external factors, also impacts neurodegenerative diseases. Inflammatory mediators can induce neuronal inflammation through neural and humoral pathways, leading to brain damage (Marizzoni et al., 2023). While peripheral lipopolysaccharide (LPS) and pro-inflammatory cytokines typically do not cause widespread neuronal damage, AD is an exception. The blood–brain barrier (BBB) becomes more permeable with age, particularly in the hippocampus, allowing peripheral inflammatory factors to activate microglia and increase central pro-inflammatory factors. In postmenopausal women, elevated systemic inflammatory factors such as TNF-*α* (95% CI 0.46 to 2.44, *p* = 0.005), IL-1β (95% CI 1.35 to 16.26, *p* = 0.02), and IL-6 (95% CI 0.06 to 1.98, *p* = 0.003) are strongly associated with increased FSH levels (Abildgaard et al., 2020). FSH induces the expression of these cytokines, contributing to both peripheral and central inflammation (Lai et al., 2022).

### 3.2 Lipid accumulation

Recent studies have highlighted the synergistic effects of FSH and apolipoprotein E4 (ApoE4) (Xiong et al., 2023a), the primary genetic risk factor for AD, in activating the C/EBPβ/*δ*-secretase pathway, which promotes AD-like pathologies (Figure 1). Ovariectomized mice mimicking a menopausal state developed AD-like pathologies, primarily driven by FSH rather than estrogen. ApoE4-knockin female mice also exhibited AD-like pathologies with increasing FSH levels, which were alleviated by anti-FSHβ antibody (FSH-Ab) treatment. Additionally, ApoE4-expressing mice showed impaired cerebrovascular integrity, elevated astrocyte hyperplasia, and disrupted BBB function, all of which accelerate AD pathogenesis (Liu C. C. et al., 2022).

Many of the proteins found in Alzheimer’s plaques have been hypothesized to be ligands for low-density lipoprotein receptors on neurons in the central nervous system (CNS), thus, FSH has been implicated in lipid deposition in neurons, leading to amyloid plaque formation (Bowen et al., 2000). A cross-sectional study found a correlation between FSH levels and cholesterol levels in women over 55 (Zhang W. et al., 2023). In mouse models, compared to the control group, high-dose FSH treatment increased serum levels of total cholesterol (TC) (1.25-fold, *p* < 0.01) and low-density lipoprotein cholesterol (LDL-C) (1.4-fold, *p* < 0.05) (Guo et al., 2019). Elevated LDL-C levels are associated with a higher incidence of AD (Saiz-Vazquez et al., 2020), with each 1 mmol/L increase in LDL-C linked to an approximately 17% increase in AD risk (Wee et al., 2023). High-density lipoprotein (HDL) facilitates Aβ transport and reduces Aβ accumulation in vascular tissue (Poliakova and Wellington, 2023). In conclusion, cholesterol may bind to lipoprotein receptor-related protein-1 (LRP1), promoting Aβ deposition and removal, vascular stiffness, arteriosclerosis, and cerebral amyloid angiopathy (CAA). Cholesterol also plays a structural role in cell membranes, which are major components of basic synaptic integrity and neurotransmission.

### 3.3 Vascular alterations

Cerebrovascular changes are another critical factor in AD. A large autopsy-based neuropathological study has revealed that 80% of AD patients without vascular dementia exhibit vascular lesions, including arteriosclerosis and CAA (Sweeney et al., 2019). Arterial stiffness is strongly associated with AD (Cortes-Canteli and Iadecola, 2020), as it reduces cerebral blood flow (CBF) and promotes Aβ deposition (Apatiga-Perez et al., 2022), involving in angiostarch deposition in early. Postmenopausal women show an inverse correlation between FSH levels and vascular compliance, with FSH contributing to vascular stiffness and endothelial damage (Laakkonen et al., 2021; Wenner et al., 2024). Notably, FSH levels were positively associated with the augmentation index (95% CI 0.68 to 1.09, *p* < 0.001) (Laakkonen et al., 2021). Path analysis further demonstrated that the effect of age on flow-mediated dilation (*p* = 0.01) was partially mediated by FSH (Wenner et al., 2024), suggesting a hormonal role in age-related vascular deterioration. FSH regulates the expression of vascular cell adhesion molecule 1 (VCAM-1) in endothelial cells through the FSHR/Gas/cAMP/PKA and PI3K/Akt/mTOR/NF-κB pathways, leading to vascular calcification and reduced elasticity (Piao et al., 2022). High FSH concentrations also disrupt the expression of V-cadherin and E-cadherin, increasing membrane permeability (Rocca et al., 2024). These vascular changes may contribute to the increased risk of AD in perimenopausal and postmenopausal women.

## 4 Blood–brain barrier breakdown

The BBB is composed of brain endothelial cells connected by tight junctions and brain perivascular cells, which include pericytes, astrocytes, microglia, and oligodendrocytes. The BBB can shield deleterious waste metabolites and remove Aβ, thereby maintaining brain homeostasis and protecting neurons (Takata et al., 2021).

**Figure 2:**
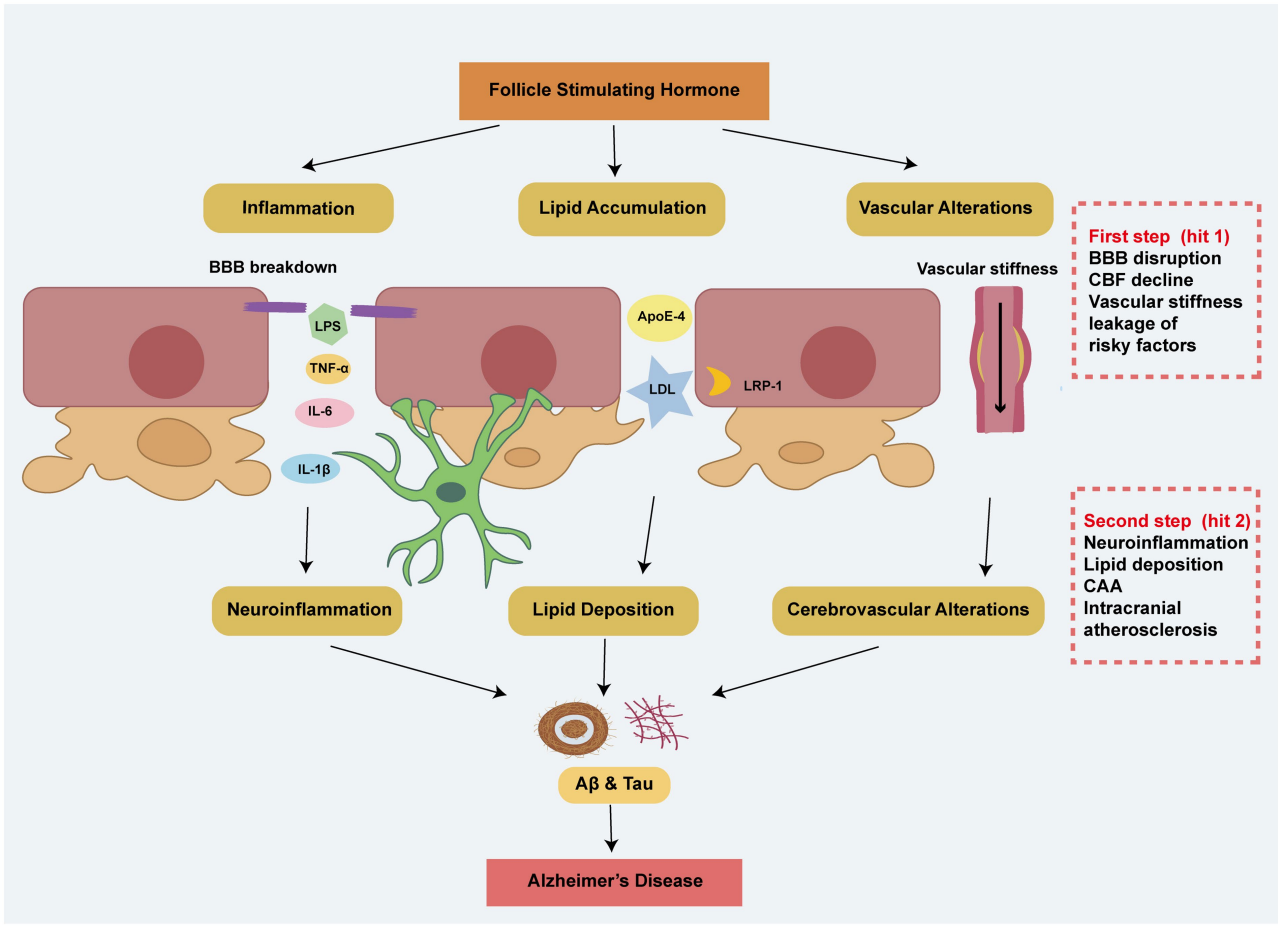
The two-hit hypothesis explains the effects of peripheral risky factors including FSH in AD. In the first step, FSH contributes to BBB breakdown, vascular stiffness, and a decline in CBF, leading to the leakage of risk factors through mechanisms involving inflammation, lipid accumulation, and vascular alterations. Building on the step 1, these peripheral risk factors exacerbate neuroinflammation, cerebral lipid deposition, CAA, and intracranial atherosclerosis, ultimately resulting in Alzheimer’s disease-like pathology.

Due to impairment of the BBB, there seems to be a close relationship between peripheral FSH, proinflammatory factors, lipids, vascular stiffness and the onset of AD. The two-hit hypothesis provides a compelling explanation for this phenomenon (Figure 2). The first hit involves BBB disruption and reduced CBF, leading to vascular-mediated neuronal dysfunction and the leakage of harmful metabolites, including FSH, lipids, and pro-inflammatory factors. The second hit involves the pathological accumulation of these risk factors, causing neurodegeneration (Eisenmenger et al., 2023). FSH may affect the cerebrovascular system on both sides of the BBB, highlighting the importance of the cerebrovascular system as a bridge between the brain and the body.

Currently, while evidence regarding the direct effects of FSH on the blood–brain barrier (BBB) remains insufficient, several interconnected mechanisms may have affection. FSH regulates the expression of connexin 43 (Cx43) in postmenopausal women (Wilson et al., 2008), potentially disrupting gap junction communication critical for BBB maintenance. The glucose transporter GLUT1, highly expressed in brain microvascular endothelial cells forming the BBB, mediates glucose transport to maintain neuronal function and BBB integrity (Koepsell, 2020), whereas FSH regulates GULT1 expression via the HIF-1α-AMPK signaling pathway (Wu et al., 2022). Additionally, VCAM-1 expressed by cerebral microvascular endothelial cells promotes cerebral vascular inflammation and damages BBB (Salian et al., 2024). While FSHR expression has been detected in peripheral endothelial cells and pericytes but not yet confirmed in brain endothelial cells (Maclellan et al., 2018). Importantly, recent studies demonstrate that FSH stimulates VCAM-1 production in vascular endothelial cells via FSHR-dependent mechanisms, enhancing monocyte-endothelial adhesion (Li et al., 2017). These findings suggest that elevated FSH levels during menopause may accelerate AD pathogenesis through combined effects on endothelial metabolism, cell–cell communication, and neurovascular inflammation.

## 5 Potential synergistic effects of FSH with other risk factors

**Figure 3:**
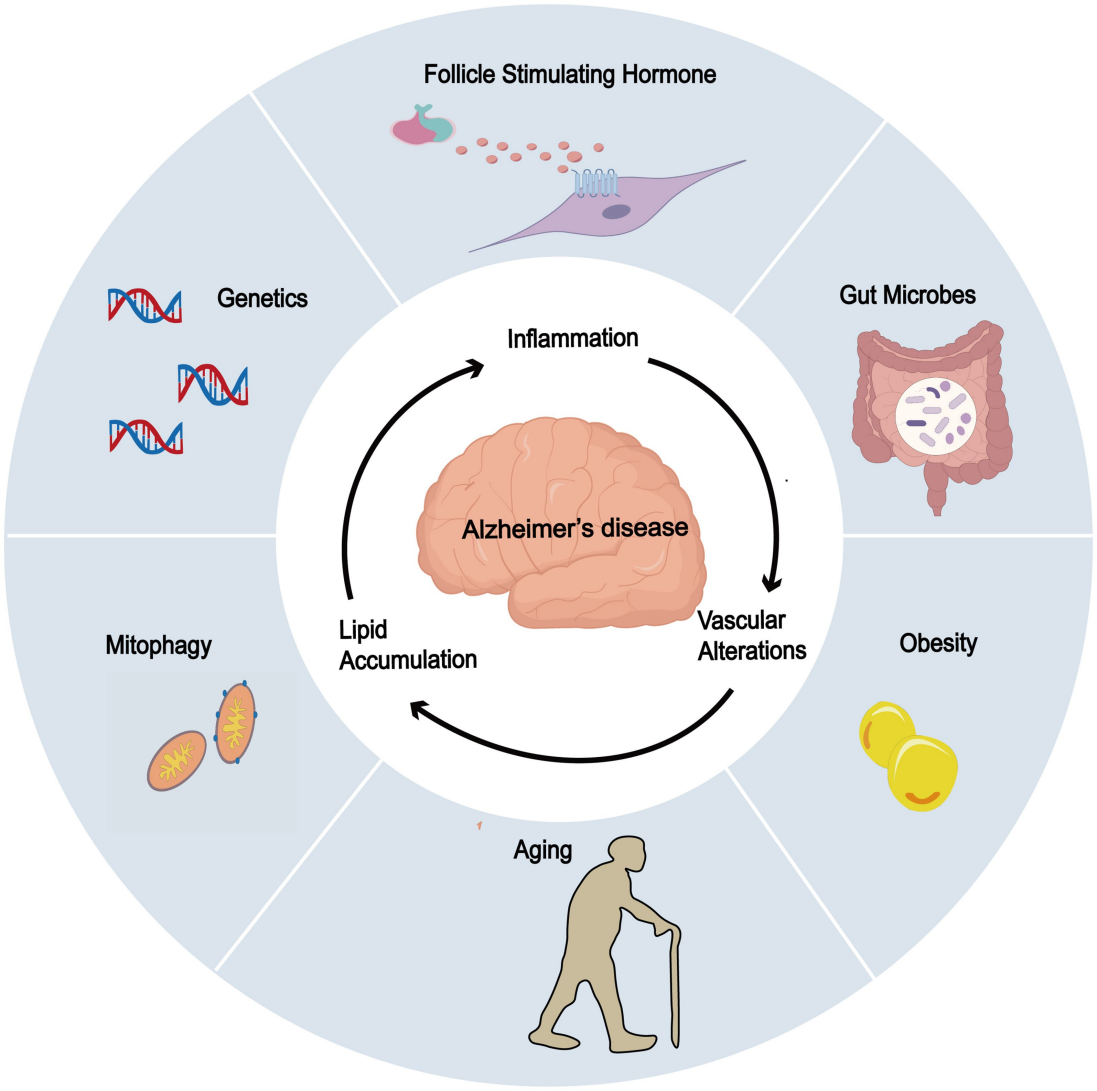
Potential association of FSH and other risk factors in AD. FSH exhibits potential synergistic effects with obesity, gut microbiota dysbiosis, impaired mitophagy, genetic factors (ApoE), and aging, all of which contribute to the development and progression of AD.

Other risk factors may synergize with FSH to create a permissive environment for the development of age-related diseases, especially AD (Figure 3).

### 5.1 Obesity

Obesity is a significant risk factor for AD in perimenopausal and postmenopausal women. Obesity impairs endothelial function, disrupts the BBB, and induces adipose tissue inflammation, leading to elevated levels of adipokines and free fatty acids (FFAs) that promote amyloid and Tau aggregation (Kao et al., 2020). FSH levels are positively correlated with body mass index (BMI) and regulate fat accumulation through the Gαi/Ca2+/CREB pathway and Ucp1 expression in visceral adipose tissue (Xiong et al., 2023b).

### 5.2 Gut microbiota

The brain-gut-microbiota axis has been increasingly studied in relation to AD. Dysbiosis of gut microbiota leads to an imbalance between pro-inflammatory and anti-inflammatory bacteria, contributing to peripheral inflammation in patients with cognitive dysfunction and cerebral amyloidosis. FSH levels are positively correlated with pro-inflammatory gut bacteria such as Escherichia/Shigella and negatively correlated with anti-inflammatory bacteria such as Eubacterium and Faecalibacterium (Kwon et al., 2023). Patients with AD-related cognitive impairment show higher levels of Firmicutes and Proteobacteria, consistent with FSH’s effects on gut microbiota (Liu Y. et al., 2022). Despite knowledge about the associations between FSH and gut microbiota, the exact mechanisms and their role in AD have not yet been fully elucidated.

### 5.3 Mitophagy

Mitophagy, the selective degradation of mitochondria, plays a crucial role in neurodegenerative diseases (Quinn et al., 2020). Increased PINK1-Parkin expression is associated with reduced Aβ levels and improved cognitive function in mice (Han et al., 2020). FSH inhibits oxidative stress in granulosa cells through the FSH-HIF-1α-PINK1-Parkin pathway (Li et al., 2020). mTOR, a downstream factor of FSH signaling, regulates autophagy. Inhibition of mTOR with rapamycin enhances autophagy, reduces Aβ levels, and alleviates AD progression.

### 5.4 Aging

In perimenopausal and postmenopausal women, aging is always the initial factor in the development of more common diseases. Chronic inflammation, dysbiosis, and impaired autophagy are emerging hallmarks of aging that interact synergistically to promote disease (Lopez-Otin et al., 2023). Moreover, during aging, the relationship between changes in hormone levels (including FSH) and pathological changes is not singular but rather involves multiple correlations. The aging of neuronal cells progressively worsens the intracranial environment, characterized by the accumulation of oxygen radicals, impaired BBB integrity, and the loss of functional synapses. These changes may drive or sustain brain inflammation by increasing the expression of inflammatory molecules, ultimately contributing to the development of AD (Guerrero et al., 2021).

## 6 Therapy and perspectives about FSH-blocking antibody

FSH has emerged as a promising therapeutic target for diseases in perimenopause and postmenopause. Antibodies targeting the *β*-subunit of FSH (FSH-Ab) have shown potential in alleviating disease progression (Table 3).

**Table 3:** Applications and functions of FSH-blocking antibodies.

FSH-Ab	Tissue	Species	Functionality	Reference
FSH-Ab	Adipose tissue	Mouse	Reduced body weight, fat mass and fat volume	Liu et al. (2017)
FSH-Ab	Serum	Mouse	Reduce the serum TC and LDL-C levels	Guo et al. (2019)
FSH-Ab	Liver	Mouse	Reduce the liver TC levels	Guo et al. (2019)
FSH-Ab	Hippocampus	Mouse	Reduction in activation of signaling pathway, formation of Aβ and Tau	Xiong et al. (2022)
FSH-Ab	Hippocampus	Mouse	Blocked the triggering of the signaling pathway, the elevation of Aβ and Tau and synapses decrease	Xiong et al. (2023a)
Mf4	Bone	Mouse	Increased bone mass	Ji et al. (2018)
Hf2	Bone	Mouse	Increased bone mass	Ji et al. (2018)
Hu26	Bone	Mouse	Inhibited osteoclast formation	Gera et al. (2020)
Hu28	Bone	Mouse	Inhibited osteoclast formation	Gera et al. (2020)
Hu6	Bone	Mouse	Inhibited osteoclast formation	Gera et al. (2020)
Hu6	Fibroblast cell	Mouse	Reversed the inhibition of Ucp1	Gera et al. (2020)
MS-Hu6	Bone	Mouse	Stimulated new bone formation and increased bone mass	Gera et al. (2022)

Early experiments demonstrated that FSH-Ab reduced fat mass and abdominal fat in mice without affecting total body weight (Liu et al., 2017). FSH-Ab also lowered serum TC and LDL-C levels in ovariectomized mice (Guo et al., 2019). In Alzheimer’s mice, FSH-Ab also inhibited the formation of plaques and neurofibrillary tangles and reversed cognitive decline (Xiong et al., 2022). Further studies revealed that FSH-Ab blocked the activation of the C/EBPβ/*δ*-secretase signaling pathway and reduced the levels of Aβ and Tau in ApoE4-TR mice. Additionally, FSH-Ab mitigated the extensive astrogliosis and microglia activation induced by ovariectomy, thereby rescuing impaired learning and memory (Xiong et al., 2023a). Monoclonal antibodies targeting FSHβ, such as Hf2 and Mf4, have been shown to increase bone mass by inhibiting osteoclast activity (Ji et al., 2018). Humanized antibodies (Hu6, Hu26, Hu28) bind FSH with high affinity and block FSHR activation, reducing osteoclast formation and promoting beige adipose tissue formation (Gera et al., 2020). New evidence also indicated that MS-Hu6 could stimulate new bone formation and increase bone mass (Gera et al., 2022). Additionally, formulated MS-Hu6 demonstrated improved stability and enhanced binding affinity to FSH at higher concentrations (Rojekar et al., 2023).

While FSH-blocking antibodies have shown promise in preclinical studies, they have yet to be applied in clinical practice.

## 7 Conclusion

This review summarizes the current understanding of the mechanisms and risk factors underlying AD in perimenopausal and postmenopausal women. FSH contributes to AD pathogenesis through neuronal signaling pathways, inflammation, lipid accumulation, and vascular alterations. The two-hit hypothesis provides a framework for understanding how peripheral risk factors, including FSH, influence AD development. Synergistic interactions between FSH and other risk factors, such as obesity, gut microbiota, autophagy, and aging, further exacerbate AD progression. A deeper understanding of the role of FSH in AD may lead to improved diagnostic methods and novel therapeutic strategies. FSH-blocking antibodies represent a promising avenue for AD treatment, though further research is needed to translate these findings into clinical applications.
